# Mouse models of myocardial infarction: comparing permanent ligation and ischaemia-reperfusion

**DOI:** 10.1242/dmm.046565

**Published:** 2020-11-18

**Authors:** Carla De Villiers, Paul R. Riley

**Affiliations:** 1 Department of Physiology, Anatomy and Genetics, University of Oxford, Oxford OX1 3PT, UK; 2 British Heart Foundation Oxbridge Centre of Regenerative Medicine, University of Oxford, Oxford OX1 3PT, UK

**Keywords:** Myocardial infarction, Mouse models, LAD ligation, Ischaemia-reperfusion

## Abstract

Myocardial infarction (MI) is a disease of major consequence in the modern world, causing permanent, irreversible damage to the heart. Survivors are at risk for developing further cardiovascular pathologies such as heart failure. Further study of MI injury is crucial to improve the understanding and treatment of the post-MI heart. The most commonly used model for MI *in vivo* is surgical ligation of the left anterior descending coronary artery (LAD). There are two predominant approaches: permanent ligation (PL), where the LAD is permanently occluded with a suture, or ischaemia-reperfusion (IR), where the LAD is temporarily occluded before removing the suture to restore blood flow and tissue reperfusion. PL results in the majority of the area at risk becoming infarcted, leading to significant apoptotic cell death and a large scar. Conversely, IR salvages some of the area at risk; thus, the scar is smaller and includes reperfusion injury, an additional, albeit smaller, second wave of necrotic damage. PL may be a more appropriate model choice for studies of heart tissue injury and wound healing, owing to the larger, more consistent infarcts, while IR enables the study of reperfusion injury. Both are clinically relevant, and the choice of model depends upon the precise pre-clinical research questions to be addressed.

## Abstract (precis)

**Summary:** Permanent ligation and ischaemia-reperfusion are common models for studying myocardial infarction. Here, we summarise the differences between them and outline the strengths of each in addressing distinct questions related to the human condition.

## Introduction

Cardiovascular disease has long been recognised as the leading cause of death globally, accounting for an enormous economic burden and significantly reducing quality of life in those affected (Virani et al., 2020). A major contributor to mortality is myocardial infarction (MI). MI is defined as an irreversible injury to the myocardial tissue due to prolonged conditions of ischaemia and hypoxia. Adult myocardial tissue lacks regenerative capacity, and, as such, injuries are permanent, leading to replacement fibrosis and permanent remodelling of the heart (Roger, 2013). Thus, despite improving survival rates for acute MI, incidence rates of chronic heart failure are on the rise. The study of the mechanisms behind ischaemic injury responses is crucial for furthering our understanding of cardiovascular pathology.

Investigation into human MI is limited by the lack of available infarcted tissue; thus, bespoke animal models are of great value to researchers. There are several available methods to model MI in animals, but the most widely used involve surgical ligation of the left anterior descending coronary artery (LAD). These models faithfully recapitulate the human responses to acute tissue injury, as well as the progression to congestive heart failure (Bayat et al., 2002). Initially developed in larger animals, advancements in technology have made LAD surgery on small animals such as rodents, and in particular the mouse, more feasible. Although the small size of the mouse adds to the technical difficulty of the procedure, there are significant advantages. Mice are relatively cheap, with high turnover rates and an abundance of genetically modified strains available to researchers. This has collectively resulted in surgical coronary artery ligation in the mouse becoming a leading procedure for the study of MI (Virag and Lust, 2011).

While initially implemented as a technique of permanent ligation (PL), an alternative method was subsequently developed, wherein the artery occlusion is temporary, allowing the study of reperfusion injury. Both versions of the LAD technique are widely used today. Several studies have compared PL and ischaemia-reperfusion (IR) injury in the mouse; however, to date, there has been no comprehensive review of the strengths and weaknesses of these techniques, nor evaluation of how each compares to the human MI. Here, we summarise the available literature on these techniques, and assess their value as surgical animal models of MI.

## PL

**Fig. 1.**
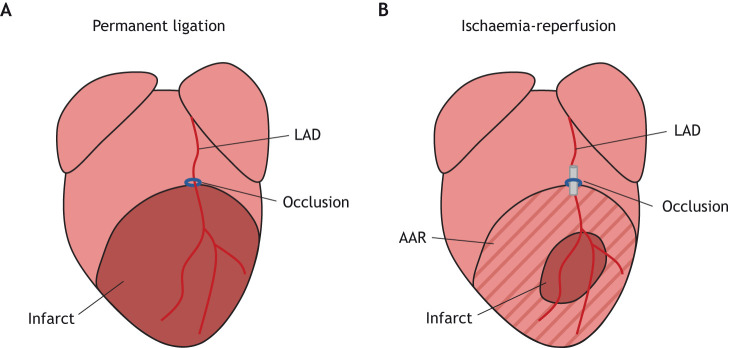
**Comparison of the infarcts generated after permanent ligation and ischaemia-reperfusion techniques.** (A) Permanent occlusion of the left anterior descending coronary artery (LAD) as a model of myocardial infarction (MI) leads to a large, permanent scar within the left ventricle. (B) Temporary occlusion of the LAD to model MI results in the restoration of blood flow and salvage of some of the area at risk (AAR), leading to a smaller infarct compared to that after permanent occlusion. The infarct size is highly variable, depending on factors such as operator experience, mouse strain, sex and period of ischaemia.

The PL procedure has been used in mice for over 60 years and, in general, aside from technical refinements, has changed little during this time (Johns and Olson, 1954; Kogan et al., 1977; Zolotareva and Kogan, 1978). Briefly, a left-sided thoracotomy is performed on an anaesthetised mouse, and a ligation is made to the left ventricle, occluding the LAD. The permanent LAD occlusion results in complete blockade of blood flow and irreversible hypoxia, which in turn results in the majority of the area at risk (AAR) becoming infarcted and a large, permanent scar (Fig. 1A). This scarred region is then prone to pathological remodelling and ultimately contributes towards progression to heart failure. The average injury size can vary widely according to mouse age, sex and, particularly, strain. In addition, the location of vessel occlusion is a key determinant in infarct size, with occlusion closer to the base of the heart, resulting in larger, more severe injury. The use of an established protocol and experienced operator minimises location-based variation in infarct sizes. In general, PL results in a large infarct, and the average infarct size tends to lie between 30% and 40% of the myocardium area, with examples summarised in Table 1 (Salimova et al., 2019). Of note, larger infarct sizes correlate, as one would expect, with lower survival rate, owing to increased chance of acute heart failure or ventricular rupture (Gao et al., 2000; Salimova et al., 2019). The survival rate also varies widely and is affected by several factors, with the genetic background of the mouse strain being the major contributing element. Poor survival has been attributed to a missense single-nucleotide polymorphism in *Cep128*, a gene involved in cilia formation, which leads to weakened heart wall strength and increased risk of myocardial rupture. This variant affects several commonly used mouse strains, including C57/Bl6. Thus, PL survival rates as low as 50% are not uncommon (Gao et al., 2010; Salimova et al., 2019).

**Table 1.**
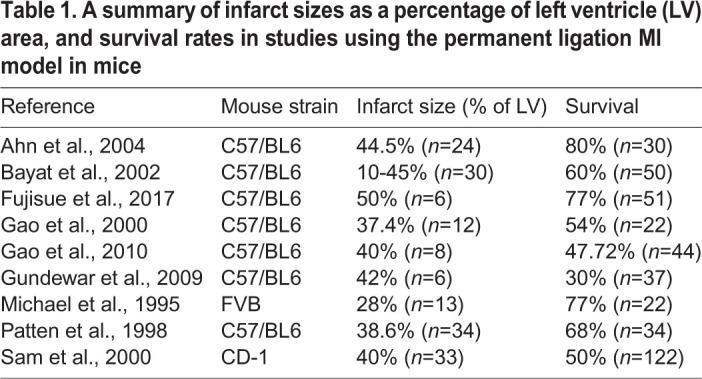
**A summary of infarct sizes as a percentage of left ventricle (LV) area, and survival rates in studies using the permanent ligation MI model in mice**

The cause of cell injury and death following PL is the extreme hypoxia within the AAR. The sudden lack of available oxygen blocks aerobic metabolism and breaks the electron transport chain, causing a rapid depletion of ATP in the cardiomyocytes. This is exacerbated by a switch to ATP hydrolysis in the mitochondria to maintain mitochondrial membrane potential. Anaerobic metabolism leads to a build-up of lactate, thus reducing intracellular pH levels. To compensate, the Na^+^/H^+^ exchanger extrudes protons, leading to an influx of Na^+^ ions and an accumulation of Ca^2+^ in the cytosol. As a result, the ischaemic conditions initiate a chain of responses that drastically alter the intracellular environment. Whereas injuries from short-term hypoxia can be recovered from, long-term hypoxia, as in the case of the PL procedure, stimulates the apoptotic pathway, leading to large-scale induced cell death in the infarcted area (Anversa et al., 1998; Kajstura et al., 1996). Consequently, the permanent hypoxic conditions after PL lead to a large area of dying tissue that is remodelled into the infarct scar.

## IR

Inducing MI with IR in rodents is a relatively new approach, as the procedure was initially used experimentally in *ex vivo* organs, then developed in dogs in 1988 (Bolli et al., 1988). Similar to the PL protocol, a left-sided thoracotomy is performed on an anaesthetised mouse, but the ligation is temporary, mediated by a small piece of tubing. Once the chosen time of ischaemia has passed, the suture can be cut and tubing removed for reperfusion. In the literature describing mouse studies, the occlusion time before reperfusion varies from 15 min to 2 h, with 30 min appearing to be the most commonly adopted. However, little information is given to justify this choice. The outcome following IR varies substantially; some of this can be explained by factors such as operator experience and mouse strain, as for the PL procedure, but the length of reperfusion adds another significant level of variation and an element of unpredictability regarding outcome. Thus, after the ‘standard’ 30 min of IR, mice may present with infarct sizes as low as 4%, manifesting as mild injury with no effect on cardiac function or further pathology, or up to 30%, considered the minimum infarct size to negatively affect function (De Celle et al., 2004; Dewald et al., 2003; Gao et al., 2010; Michael et al., 1995). This, in turn, means that more mice are required per experiment, contravening the best practice of refinement, reduction and replacement (3Rs). Table 2 summarises the examples of reported infarct sizes upon IR. In all cases, the infarct size after IR is significantly smaller than in PL, because a portion of the AAR is salvaged by the restoration of blood flow (Fig. 1B).

**Table 2.**
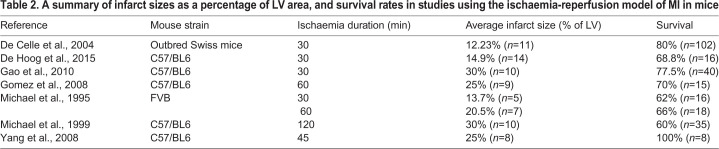
**A summary of infarct sizes as a percentage of LV area, and survival rates in studies using the ischaemia-reperfusion model of MI in mice**

One major difference between IR and PL is the secondary onset of reperfusion injury. This occurs as a direct result of the sudden restoration of blood flow to the AAR, and acts as an additional source of cell damage and death after ischaemia. As explained above, the hypoxic conditions during ischaemia result in depletion of ATP and lowering of intracellular pH. With reperfusion, oxygen becomes rapidly available, allowing oxidative phosphorylation to resume. This switch back to aerobic metabolism generates reactive oxygen species, and reperfusion is associated with a burst of free radical production, with levels remaining elevated for several hours (Bolli et al., 1988; Garlick et al., 1987; Nossuli et al., 2001). With the electron transport chain functioning once more, mitochondrial membrane potential is restored and pH normalises. Together, the oxidative stress coupled with pH restoration induce the opening of the mitochondrial permeability transition pore (Sanada et al., 2011). This large, non-specific pore allows passage between the mitochondrial matrix and the cytosol, which disrupts the normally finely regulated transport of protons and ions, and leads to swelling and rupture of the mitochondria, which triggers necrotic cell death (Jennings et al., 1960). As such, the apoptotic cell death that results from the initial ischaemic insult is accompanied by an additional wave of necrotic cell death from reperfusion injury (Kalogeris et al., 2012; Michael et al., 1995; Simonis et al., 2012).

## Comparing the utility of the techniques

The PL and IR procedures are both valuable tools for the modelling and study of MI. Although based on the same principles, PL and IR have differences that researchers should consider when choosing which model is most appropriate for the experimental question being pursued.

One major difference between the two techniques is the infarct size after surgery. Although this can vary dramatically, an experienced operator can generally expect severe infarcts after PL, whereas with the IR procedure, reperfusion salvages parts of the AAR, resulting in a smaller infarct size and a less severe injury. Mice are more tolerant of myocardial injury than humans. Infarct sizes of <30% are well tolerated in mice, and their hearts retain sufficient contractile function to prevent progression towards heart failure (Bayat et al., 2002; Sam et al., 2000). As such, many studies exclude animals for which infarct sizes are below 30%, deeming these insufficiently injured for the study of the infarcted heart (Salimova et al., 2019; Sam et al., 2000). This exclusion criterion cannot be applied to the IR technique, using which infarct size is often well below 30% after the typical 30 min of ischaemia. As such, IR infarcts are often associated with low-level remodelling that fully rescues cardiac function (De Celle et al., 2004). De Celle et al. compared the effects, in the mouse, of a 30-min ischaemia period followed by reperfusion to those after PL, 8 weeks post-injury. The average infarct size was 52% in seven PL animals and only 12.3% in 11 IR animals. No decrease in cardiac function was recorded in the IR group (De Celle et al., 2004). Longer ischaemic periods naturally result in more severe injuries, with Michael et al. showing that increasing the ischaemic period to 2 h increases IR infarct sizes to 30% – similar to those seen in the PL group in the same study (Michael et al., 1999). Interestingly, despite comparable infarct sizes, cardiac function appeared somewhat protected in the IR group compared to the PL group. Mice in the PL group revealed a steady worsening in aortic flow velocity and diastolic filling, whereas mice in the IR group showed an initial drop 1 week post-injury, followed by a rescue of function from 2 weeks onwards. This was associated with a greater degree of hypertrophy in the PL group (Michael et al., 1999). Thus, even late reperfusion provides a protective effect to the cardiac tissue post-MI, and this is corroborated by observations in clinical studies, whereby late reperfusion reduced left ventricle dilatation and remodelling (Dalen et al., 1988; Kennedy et al., 1985; Topol et al., 1992). When studying tissue responses post-MI injury in experimental models, a larger and more severe injury is usually preferred, as it gives a clearer comparison between injured and sham animals. A milder injury, such as that following IR, can mask otherwise significant changes between injured and healthy tissue. Thus, for general studies comparing injured and healthy tissue, the PL procedure might be more appropriate.

Studies comparing protein or mRNA expression in infarcted tissue post-MI have shown differences between PL and IR. However, these studies mainly detected differences in tissue injury markers, which could be caused by differences in infarct sizes between the techniques (Charles et al., 2000; Ivanov et al., 2018). In particular, a comparative proteomics study on heart tissue from PL and IR mice carried out by De Celle et al. identified several proteins with differential expression specific to each technique (De Celle et al., 2004). These included cardiac troponin T and α-tropomyosin, the expression of which increased in mice following PL compared to that in IR animals. These myofilament proteins are involved in muscle contraction, and previous work has shown that cardiac troponin T is a sensitive marker for myocardial damage, with a direct correlation between expression and infarct size (Remppis et al., 2000). Similarly, the De Celle study found expression of serum amyloid P-component precursor, which activates the complement system during inflammation, specifically increased after PL compared to IR (De Celle et al., 2004). An increase in the expression of heat shock proteins (HSPs) 20 and 27 was also reported only from PL tissue. The expression of HSPs has been previously described to increase under conditions of cellular stress, and studies in human and rat have reported increases to HSP27 in myocardium after heart failure (Knowlton et al., 1998; Scheler et al., 1999; Tanonaka et al., 2001). With previous studies by De Celle et al. reporting infarct sizes four times smaller in the IR model compared to the PL model (De Celle et al., 2004, 2005), it is likely that these PL-specific expression changes are more related to the severity of the injury than to any mechanistic differences in cellular responses and pathology between techniques. Conversely, IR caused a change in expression of annexin A3, which was decreased in cytosolic fractions and increased in the membrane (De Celle et al., 2005). Annexin A3 is a multi-functional phospholipid-binding protein involved in processes such as membrane trafficking, cell signalling and coagulation (Gerke and Moss, 2002). This translocation from the cytosol to the membrane might be a consequence of reperfusion injury; however, further studies are needed to understand the cause, effect and any downstream consequences.

## Clinical relevance

It could be argued that IR is more representative of human MI than PL. For patients presenting with MI, the front-line treatment option is timely reperfusion therapy; namely, thrombolytic therapy, balloon angioplasty or primary percutaneous coronary intervention (PPCI). Although reperfusion injury, as discussed above, is an issue that researchers need to consider when planning experiments, it remains clinically beneficial to begin reperfusion therapy as early as possible to increase AAR salvage and limit the final infarct size. Reperfusion injury is not a factor in the PL procedure, and thus the research focus is on the initial tissue injury response. However, although timely reperfusion is critical for human patients, studies have shown that a significant portion of patients receive therapy late, and 15-30% of patients are admitted to hospital too late after onset of the MI for reperfusion therapy to provide any benefit (Cohen et al., 2010; Gharacholou et al., 2010; Guan et al., 2018). Therefore, a sizeable portion of the MI patient population experience a long-term ischaemic condition, for which modelling by the PL procedure could be more relevant. In addition, the bulk of the injury after IR remains the result of ischaemia, with reperfusion injury accounting for a much smaller, second wave of post-MI injury. As such, reperfusion injury might not have a measurable effect on the overall extent of MI. However, angioplasty and PPCI treatment can cause reperfusion injury in MI patients. The mouse IR model, therefore, may enable a sufficient depth of understanding of this injury response to develop secondary therapies to mitigate reperfusion injury in MI patients.

## Conclusion

PL and IR are common models for the investigation of MI, and have so far provided significant mechanistic understanding of ischaemic cardiac pathology. Advances in surgical technique have rendered both approaches feasible in the mouse. PL produces larger, more severe infarct sizes, is associated with poor survival rate and enables the basic study of acute tissue injury, repair responses and ensuing pathologies, including progression to heart failure. PL is directly applicable to the approximate 30% of acute MI human patients who do not receive reperfusion therapy and for those that do but progress to heart failure. In contrast, IR is more technically challenging than PL, producing smaller and more variable infarcts that often do not progress into further cardiovascular pathologies, but provides the opportunity to study the second wave of injury associated with blood reperfusion, which is applicable to clinical interventions in human acute MI patients and which is currently not a therapeutic target (Box 1).

**Box 1. Translational impact**

- Cardiovascular disease is a leading cause of death globally, with myocardial infarction (MI) being the most common contributor. Animal models of MI are crucial tools to further our understanding of the disease.
- The permanent ligation (PL) technique results in a large infarct size, as the majority of the area at risk (AAR) becomes injured.
- The infarct after PL is caused by apoptosis following long-term hypoxia.
- The ischaemia-reperfusion (IR) technique causes a variable infarct size, depending on the length of ischaemia. The most common duration used in mice is 30 min, but this time point is not well justified against clinical criteria.
- Infarct sizes after IR are generally smaller than after PL, because a portion of the AAR is salvaged by reperfusion. Even after delayed reperfusion, where the infarct size is comparable to that after PL, cardiac function and remodelling post-MI is protected by reperfusion.
- The infarct after IR is caused by an initial area of apoptosis following hypoxia, as well as a smaller second wave of necrosis as a result of reperfusion injury, caused by reactive oxygen species and by the opening of the mitochondrial permeability transition pore.
- PL provides more reproducible infarct sizes for the study of repair and regeneration than IR. The use of PL models has been key for research into cell- and drug-based regenerative therapies after MI. IR is highly variable, but enables study of reperfusion and reperfusion injury. Studies in IR models have emphasised the importance of timely reperfusion in the clinical setting, and directed treatment strategies towards restoring blood flow to the site of injury. These therapies, including thrombolytics, angioplasty and primary percutaneous coronary intervention (PPCI), have led to a significant reduction in acute mortality after MI.

Both PL and IR models are key tools in the study of MI, have clinical relevance and should be employed according to the specific pre-clinical question researchers intend to address. Importantly, each has the potential to provide insight into designing future therapies for acute MI human patients. Further studies on MI are crucial to advance our understanding of acute MI in human patients, and to improve lead drug development and treatment strategies in the future. To this aim, many complementary experimental models may be useful, including *in vitro* studies on cultured cardiovascular cell types, or *ex vivo* studies using isolated perfused hearts (Lindsey et al., 2018). The use of *in vivo* models arguably provides the most translationally relevant insights into the clinical condition. As such, PL and IR serve as valuable additions to the repertoire of experimental models available for the study of MI and to advance the development of novel therapeutics.
